# New findings in a 400 million-year-old Devonian placoderm shed light on jaw structure and function in basal gnathostomes

**DOI:** 10.1038/s41598-017-07674-y

**Published:** 2017-08-10

**Authors:** Yuzhi Hu, Jing Lu, Gavin C. Young

**Affiliations:** 1 Department of Applied Mathematics, Research School of Physics and Engineering, Oliphant Building 60, Australian National University, Canberra, ACT 2601 Australia; 2 Research School of Earth Sciences, Building 142 Mills Road, Australian National University, Canberra, ACT 2601 Australia; 3 Key Laboratory of Vertebrate Evolution and Human Origins of Chinese Academy of Sciences, Institute of Vertebrate Paleontology and Paleoanthropology, Chinese Academy of Sciences, Beijing, 100044 China

## Abstract

Arthodire placoderms have been proposed as the sister group of Chinese ‘maxillate’ placoderms plus all the more crownward gnathostomes. These basal groups provide key information for understanding the early evolution of jaws. Here, we test previous assumptions about placoderm jaw structure and function by using high-resolution computed tomography, digital dissection, and enlarged 3D printouts on a unique articulated 400 million-year-old buchanosteid arthrodire. The upper jaw has a double ethmoid and a palatobasal connection, but no otic connection; the dermal bone attachment for the quadrate is different to other placoderms. A separately ossified cartilage behind the mandibular joint is comparable to the interhyal of osteichthyans. Two articular facets on the braincase associated with the hyomandibular nerve foramen supported a possible epihyal element and a separate opercular cartilage. Reassembling and manipulating 3D printouts demonstrates the limits of jaw kenetics. The new evidence indicates unrecognized similarities in jaw structure between arthrodires and osteichthyans, and will help to clarify the sequence of character acquisition in the evolution of basal gnathostome groups. New details on the hyoid arch will help to reformulate characters that are key in the heated debate of placoderm monophyly or paraphyly.

## Introduction

The extinct placoderms (‘armoured fishes’), with large dermal bones that were readily preserved as fossils, are the best-known and most diverse vertebrate group of the Devonian Period (~420–360 million years ago), and were globally distributed in all habitable aquatic environments^1^. As early gnathostomes (jawed vertebrates), placoderms were traditionally viewed as a side branch to the main trajectory of jaw evolution from early osteichthyan fishes to the first tetrapods (land vertebrates). However, the recent discoveries of the ‘maxillate’ placoderms *Entelognathus* 2 and *Qilinyu* ^3^ from the Silurian of China, combining both placoderm and osteichthyan features, have changed that scenario.

Two of the major placoderm subgroups (antiarchs and arthrodires) have been placed as basal branches of a paraphyletic stem of the gnathostome tree^2^. More recent analyses have interpolated other placoderm taxa (such as *Brindabellaspis*, petalichthyids and *Romundina*) within a paraphyletic^4–8^ or monophyletic^9^ assemblage of stem gnathostomes. The Arthrodira, comprising about 55% of some 330 named placoderm taxa^1^, included the largest marine predators of Devonian seas (e.g. the Late Devonian *Dunkleosteus* from the Cleveland Shale of Ohio). Their highly evolved blade-like dermal jaw bones have been used as exemplars in analyses of the early evolution of jaws and teeth^10–12^. However, arthrodires underwent major evolutionary changes in jaw structure during ~70 million years of their existence^1^, and jaw structure and function in basal members of the group have been poorly understood.

Our new information on jaw structure is based on an exceptionally preserved specimen of ‘buchanosteid’ arthrodire (ANU V244) from the Early Devonian limestones (~400 Ma) at Burrinjuck, near Canberra, south-eastern Australia (Supplementary Fig. 1)^13^. It had been partly etched with formic acid before Micro-CT (computed tomography) scanning. ANU V244 belongs within the family Buchanosteidae^14^, but its precise species and relation to other buchanosteids is unclear. ANU V244 shows numerous other characters unknown in most other placoderms, and is referred to in the text as a ‘buchanosteid’ (For more detail see Supplementary Information). The closely related *Buchanosteus* ^15, 16^ has been used as the basal member of brachythoracid arthrodires in recent analyses^7, 17, 18^, but key information such as the underlying jaw cartilage morphology has not been documented. Unlike later arthrodires, in which the cartilaginous endoskeleton is generally not preserved, our specimen preserves the braincase and endoskeleton of the jaws by investment of perichondral bone, permitting the first complete description of the palatoquadrate and Meckel’s cartilage for any arthrodire.

For the first time in a fossil vertebrate, we have used high-resolution 3D printing to investigate placoderm jaw morphology and function by experimentation of the morphological fit between all dermal and perichondrally-ossified endoskeletal elements of the skull, braincase, jaws and cheek.

## Results

**Figure 1:**
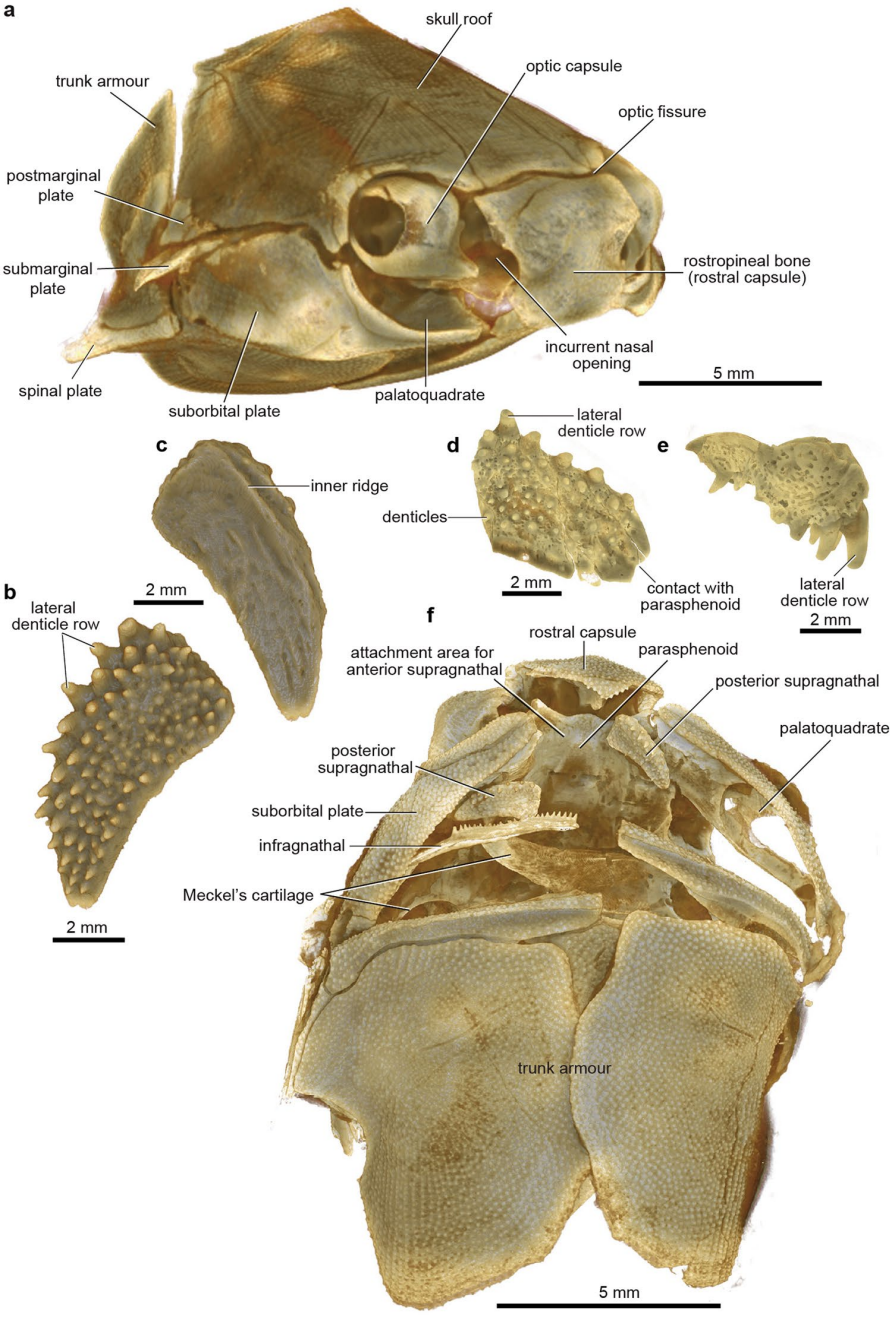
Articulated buchanosteid arthrodire (ANU V244) based on high-resolution CT. (**a**) Whole specimen in right anterolateral view. (**b**–**c**) Right posterior supragnathal bone in occlusal (**b**) and dorsal (**c**) views. (**d**–**e**) Right anterior supragnathal bone in occlusal (**d**) and lateral (**e**) views. (**f**) Whole specimen in ventral view.

**Figure 2:**
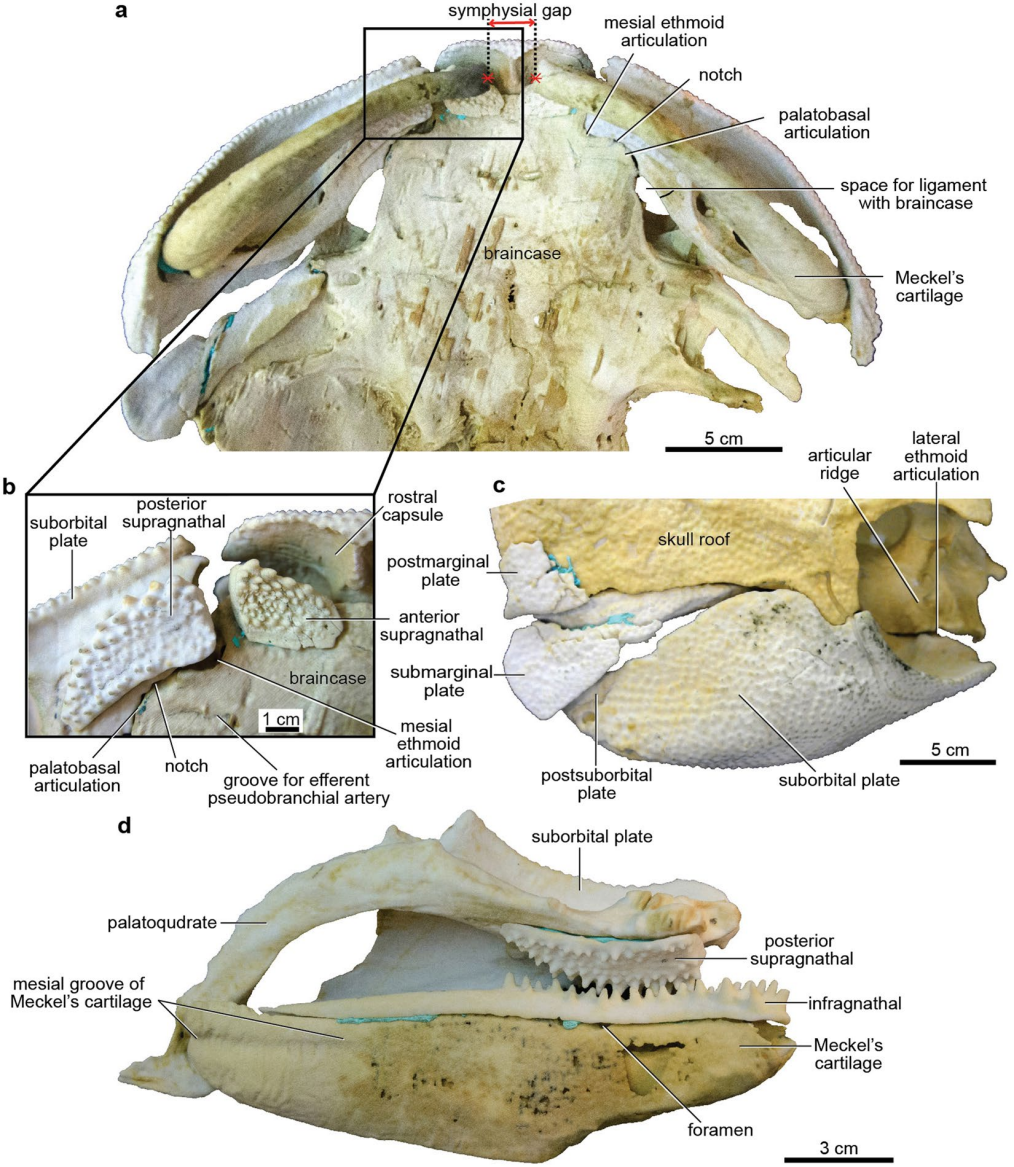
Articulated buchanosteid arthrodire (ANU V244) based on 3D printouts. (**a**) Ventral view of all jaw components in position against the braincase, showing a symphysial gap between left and right Meckel’s cartilages. (**b**) Boxed area of (**a**), with Meckel’s cartilage removed. (**c**) Dermal elements of cheek and operculum in position against the skull roof and braincase; right lateral view. (**d**) Jaw elements of left side in occlusion. 3D printouts are 6 times natural size.

The whole specimen (Fig. 1) displays a condition shown by certain basal placoderms, with the olfactory part of the braincase, and surrounding dermal bones including the pineal opening, separately ossified by an ‘optic fissure’ to form a discrete ‘rostral capsule’. The cheek unit (best preserved on the right side) comprises dermal suborbital and postsuborbital plates attached to the outside of the upper jaw cartilage (palatoquadrate). Behind this is the dermal opercular cover (submarginal plate), that was movable against the anterior edge of the dermal shoulder girdle (trunk-armour). The various jaw components are slightly displaced and preserved partly inside the ventral dermal trunk-armour (Fig. 1b–f). The jaw cartilages show complete perichondral ossification. In the original specimen these extremely fragile structures are largely obscured by external dermal bones^13^, and their detailed morphology, and experiments with gnathal plate occlusion, jaw cartilage articulation, and other functional aspects as described below, could not be investigated without the new techniques of micro-CT scanning and 3D printing (Fig. 2).

## Description

### Gnathal plates and Jaw cartilages

**Figure 3:**
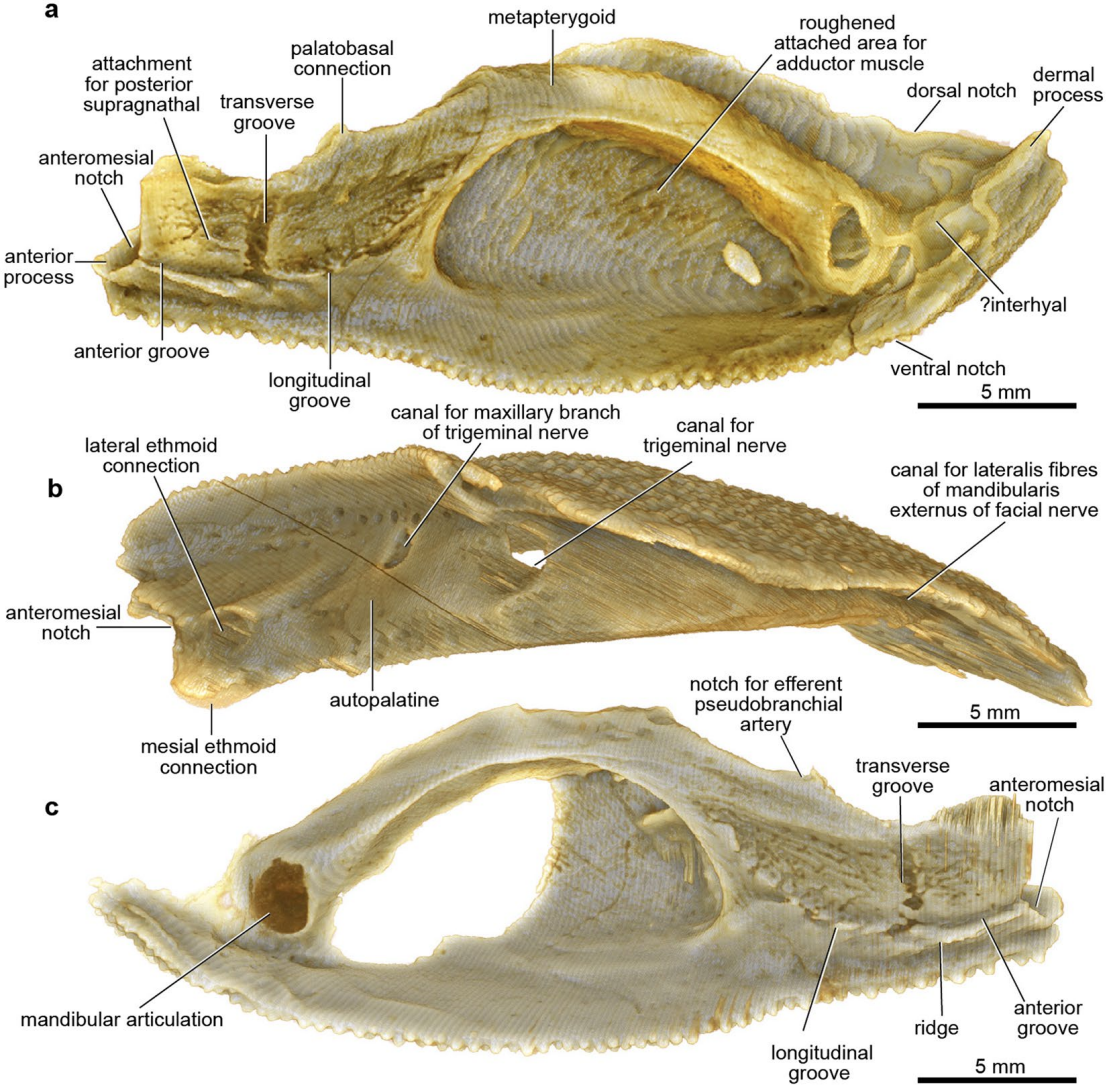
Dermal cheek unit with attached perichondrally ossified cartilage elements of articulated buchanosteid arthrodire (ANU V244) based on high-resolution CT. (**a**,**b**) Right suborbital and postsuborbital plates, with attached palatoquadrate and? interhyal, in internal (**a**) and dorsal (**b**) views. (**c**) Left suborbital plate (incomplete) and attached palatoquadrate in internal view.

Only one anterior supragnathal was preserved. It is the right element, not the left as previously interpreted^13^, when it was assumed the anterior supragnathals were in midline contact. There is a short contact face for the anterior edge of the parasphenoid (Fig. 1d). There are clearly defined attachment surfaces for both supragnathals on the palatoquadrate (posterior; Fig. 3a) and the braincase. Both have a highly-vascularized surface, and the attachment on the palatoquadrate shows distinct grooves, named anterior, transverse, and longitudinal (Fig. 3c), the last with at least six small mesial branches.

The perichondrally-ossified palatoquadrate is fused to the inside of the dermal suborbital plate (Fig. 3a). Palatoquadrate morphology generally conforms to that described from the only previously known buchanosteid palatoquadrate, which lacked the quadrate portion^15, 16^. On the autopalatine, two clear articulations formed an ethmoid connection with the braincase (Fig. 3b). The lateral articulation on its upper surface is supported by a buttress-like ridge, and filled with hard tissue. The mesial articulation is an opening as previously described (i.e. cartilage-filled in life). An anteromesial notch at the front end (Fig. 3) does not connect with the braincase when the palatoquadrate is in position (Fig. 2a,b). Farther back, a distinct process on the mesial edge of the autopalatine represents the palatobasal connection with the braincase. Previous descriptions noted a double articulation here^15, 16^, but in V244 there is only one articular facet, behind which is a notch for the efferent pseudobranchial artery (Fig. 3c), identified from the position of the groove for this artery on the ventral braincase floor.

Behind the orbit the metapterygoid section is highly arched, with only a narrow contact area with the dermal bone (Fig. 3c). A roughened area on the inner dermal surface indicates the extent of attachment of the adductor mandibulae muscle to the dermal bone (Fig. 3a). The lateral part of the muscle would have inserted here, and the mesial adductor fibres would have attached on the palatoquadrate, but still on the lateral face of the metapterygoid, thus conforming with the fundamental relationship of the adductor mandibulae muscle lateral to the upper jaw cartilage in all fishes^15^. In the arthrodire *Dicksonosteus*, the restored adductor muscle passes from the lateral side of Meckel’s cartilage to the mesial side of the palatoquadrate, which could suggest a major difference in adductor mandibulae musculature between placoderms and other gnathostomes^19^. However, closure of the adductor fossa by a lateral lamina of the palatoquadrate in *Dicksonosteus* is considered a derived state relative to the condition in other placoderms^16, 20^. The ventral adductor embayment in various other placoderms such as *Holonema* ^21^, *Romundina* ^22^, *Bothriolepis* ^23^, and *Nefudina* ^24^, with adductor insertion mainly on the outer face of the palatoquadrate (between it and the dermal bone), corresponds to the situation in our buchanosteid specimen. For ‘*Buchanosteus*’ this feature was previously incorrectly coded^25^, but has been subsequently updated (character 47)^2^.

The dorsal view of the palatoquadrate (Fig. 3b) shows the openings of three large canals passing between the cartilage and the dermal bone. As previously interpreted^15^, the anterior two evidently carried maxillary and mandibular branches of the trigeminal nerve (V), and probably also branches of the ramus buccalis lateralis of the facial nerve (VII). The posterior opening would have transmitted lateralis fibres (ramus mandibularis externus VII) supplying most of the sensory line grooves on the exterior of the dermal cheek plates (Supplementary Fig. 4). Apart from the infraorbital groove, all other sensory grooves and pits of the cheek in fishes are supplied by this lateralis branch from the hyomandibular nerve^26^. The canals that transmitted these nerves all lie between the dermal bone and the body of the palatoquadrate, and at no point enter the latter. This is evidently a simpler relationship than restored for *Dicksonosteus* ^27^, in which the maxillary branch of V and the buccalis lateralis of VII are shown passing mesial to the palatoquadrate before emerging on the lateral side of the autopalatine. In our specimen, these nerves again show the fundamental position for all jawed fishes^15^, as demonstrated in modern dissections (e.g. *Polypterus* ^28^; *Chlamydoselachus* ^29^), by being entirely external to the palatoquadrate, and presumably also to the adductor mandibulae muscle.

One previous specimen of the buchanosteid palatoquadrate was described, but the quadrate portion was broken off^15^. In ANU V244 this region is well preserved, the articular surface for the mandibular joint being a distinct oval-shaped opening, partially broken on the right side, but with its margins complete on the left (Fig. 3c). The quadrate is entirely attached to the inside of the suborbital plate, contrary to previous assumptions^15, 16^, and the situation in other arthrodires^21, 30^, where the quadrate is fused inside the postsuborbital plate (Supplementary Fig. 4). In ANU V244 the position of the dermal suture is indicated by dorsal and ventral notches, and a completely separate perichondral ossification, attached inside the postsuborbital plate (Fig. 3a), is positioned just behind the quadrate, inviting comparison with the interhyal of osteichthyans. This separate ossification does not contact the quadrate. It has a slight rounded dorsal process, and a mesial protuberance that possibly connected with another hyoid arch element. Posterodorsally, it is continuous with a pointed dermal process projecting above the edge of the postsuborbital plate (Fig. 3a). Reassembling the cheek unit using 3D printouts shows that this process fitted under the edge of the submarginal plate (Figs 5b,c and 6b). This would have interlocked the cheek and operculum, and limited the opercular opening. We note that the holotype of *Dicksonosteus* may have had a similar arrangement, with the right postsuborbital plate clearly behind the palatoquadrate (slightly displaced), but suggesting a similar pointed dermal process projecting up inside the submarginal plate^31^.

**Figure 4:**
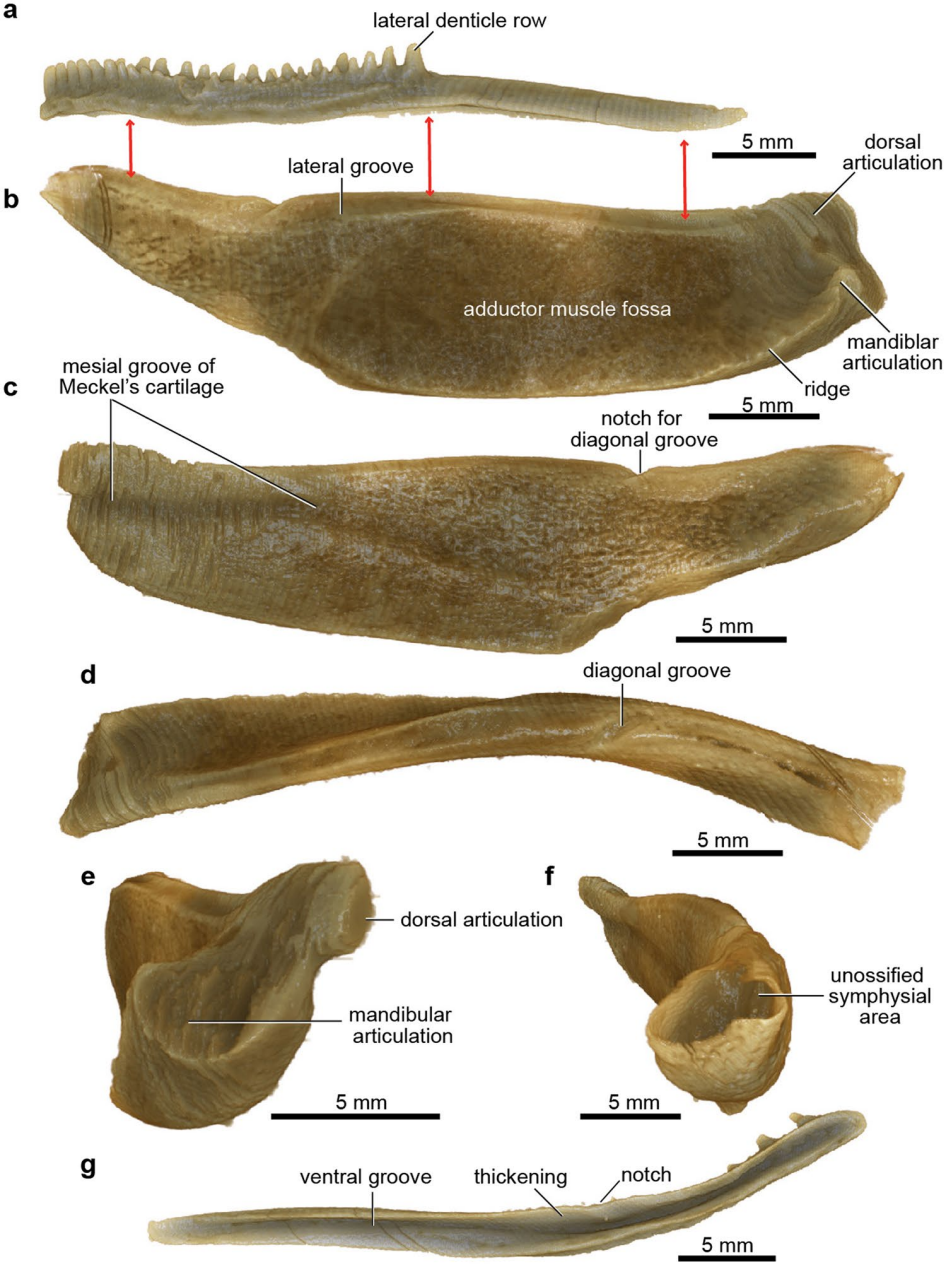
Lower jaw elements of articulated buchanosteid arthrodire (ANU V244) based on high-resolution CT. (**a**) Infragnathal in left lateral view. (**b**-**f**) Meckel’s cartilage in left lateral (**b**), mesial (**c**), dorsal (**d**), posterior (**e**), and anterior (**f**) views. (**g**) Left infragnathal in ventral view (reversed image of extracted right element).

For the lower jaw, the infragnathal bone and Meckel’s cartilage of both sides are preserved. The right infragnathal bone (the left previously removed and restored^13^), and both Meckel’s cartilages are slightly displaced posteriorly in the specimen (Fig. 1f; Supplementary Fig. 2c). The infragnathal is a slender dermal bone with an anterior denticulate biting portion carrying two main longitudinal rows of denticles, and a slightly shorter posterior non-biting portion (Fig. 4a). A deep ventral groove (Fig. 4g) fitted over the dorsal edge of Meckel’s cartilage in life. Meckel’s cartilage is completely ossified perichondrally as a single element (Fig. 4b–f), a condition unrecorded for any other arthrodire. The digitally extracted left element is deepest in its anterior half, whereas the corresponding element in sharks (e.g. *Gogoselachus* ^32^) is generally deepest posteriorly. A distinct lateral groove along the dorsal edge (Fig. 4b), not previously recognized^13^, indicates that the infragnathal extended farther back than previously restored, right to the mandibular joint, as demonstrated by 3D printouts (Fig. 2d). This also shows the massive size of Meckel’s cartilage relative to the slender palatoquadrate. In contrast, most Palaeozoic elasmobranchs have the palatoquadrate of similar size or relatively larger^33^. This difference is likely due to the added strength to the upper jaw provided by the large dermal bones in placoderms.

The dorsal surface of Meckel’s cartilage is notched by a diagonal groove (Fig. 4c), with a ventral thickening and notch in the corresponding position on the infragnathal (Fig. 4g). The restored lower jaw using 3D printouts (Fig. 2) shows a foramen opening into a canal that transmitted a nerve or vessel passing forwards from the mesial to the lateral side, between the dermal bone and the cartilage. Other new features of Meckel’s cartilage include a ridge defining the posteroventral edge of the adductor muscle fossa on the lateral surface (Fig. 4b), also recently identified in *Gogoselachus* ^32^, and a mesial groove running forward from the notch between the two posterior articular surfaces (Fig. 4c). This groove extends much farther forward than previously reconstructed, with its anterior end deflected ventrally to the ventral edge of the cartilage. It may have carried the internal mandibular branch of the trigeminal nerve^13^.

### Mandibular joint

**Figure 5:**
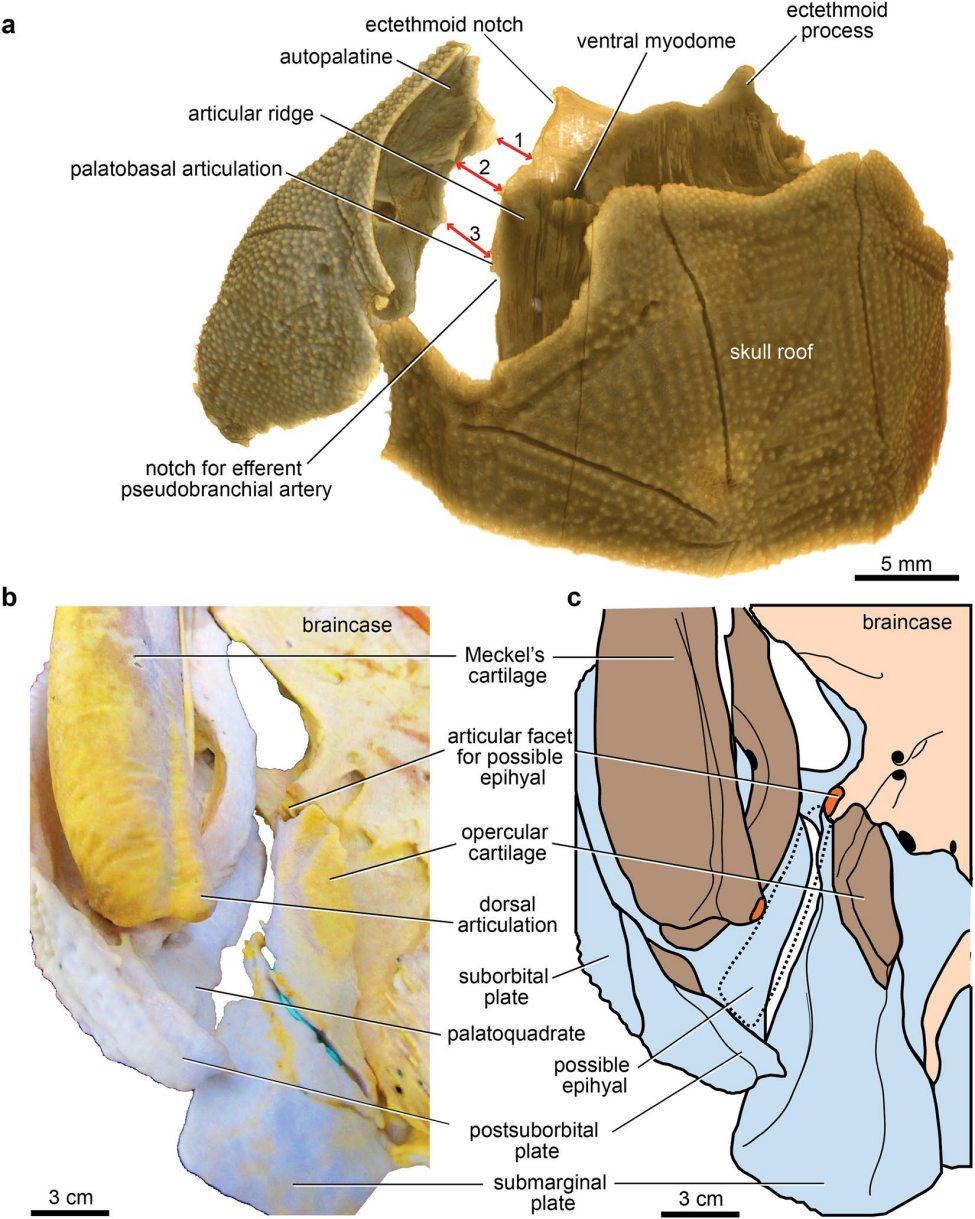
Jaw articulations of articulated buchanosteid arthrodire (ANU V244) based on high-resolution CT. (**a**) Dorsal view summarising articulations between the left autopalatine and subnasal shelf of the braincase (red arrows: 1, mesial articulation of ethmoid connection; 2, lateral articulation of ethmoid connection; 3, palatobasal connection). (**b**) Ventral view of assembled 3D printouts of right Meckel’s cartilage, palatoquadrate, suborbital and postsuborbital plates in position against the skull and braincase. (**c**) Reconstruction based on (**b**). Light blue, dermal bones; brown, visceral arch cartilages; pale pink, braincase; orange, articular facets. 3D printouts are 6 times natural size.

Assembling 3D printouts of the jaw components (Fig. 2; Supplementary Fig. 3a,b) demonstrates that the larger ventral articular surface on Meckel’s cartilage formed the mandibular joint (Fig. 4e), not the dorsal articulation as previously interpreted^1, 13^. The unossified mandibular articulation on the palatoquadrate (Fig. 3c) presumably contained a convex cartilaginous articular surface, given the concave shape of the corresponding articulation on Meckel’s cartilage. Placing 3D printouts of the palatoquadrate and Meckel’s cartilage together, with dermal gnathal elements (posterior supragnathal, infragnathal) in occlusion (Fig. 2d), shows the space beneath the arched metapterygoid part of the palatoquadrate sitting directly opposite the adductor fossa on the lateral face of Meckel’s cartilage (Supplementary Fig. 2e). The ‘dorsal’ articular area on Meckel’s cartilage (Fig. 4e) projects mesially towards the skull and braincase (Fig. 5b,c). By comparison, in Gogo arthrodires there may be several articular areas on the quadrate and articular, but our specimen suggests that the mandibular joint may not always have been correctly identified, without the aid of reassembly using 3D printouts.

### Jaw restoration and gnathal plate occlusion

Reassembling 3D printouts of all jaw elements against the skull and braincase (Fig. 2a) clearly demonstrates several articulations between the autopalatine and the subnasal shelf of the endocranium. The ethmoid connection of the palatoquadrate comprises two (mesial and lateral) articulations, and the anteromesial notch (Fig. 3a,c) does not contact the braincase, but sits opposite an ectethmoid notch of the subnasal shelf (Fig. 5a). The mesial ethmoid articulation on the palatoquadrate fits into a large rectangular depression on the ventral surface of the subnasal shelf (Fig. 2b; Supplementary Fig. 5), a structure not shown in previous reconstructions^14, 15^. However this is in essentially the same position, and is surely homologous with the main anterior connection of the palatoquadrate in *Kujdanowiaspis* ^34^, and *Dicksonosteus* ^27, 31^ (previously this has been called an ‘orbital connection’^16^). The lateral ethmoid articulation (Fig. 3b) connects with the dorsal surface of the subnasal shelf, where it is buttressed by an ‘articular ridge’ running posterolaterally from behind the ventral myodome (Figs 2c and 5a). Again this structure was not previously identified^15, 16^.

**Figure 6:**
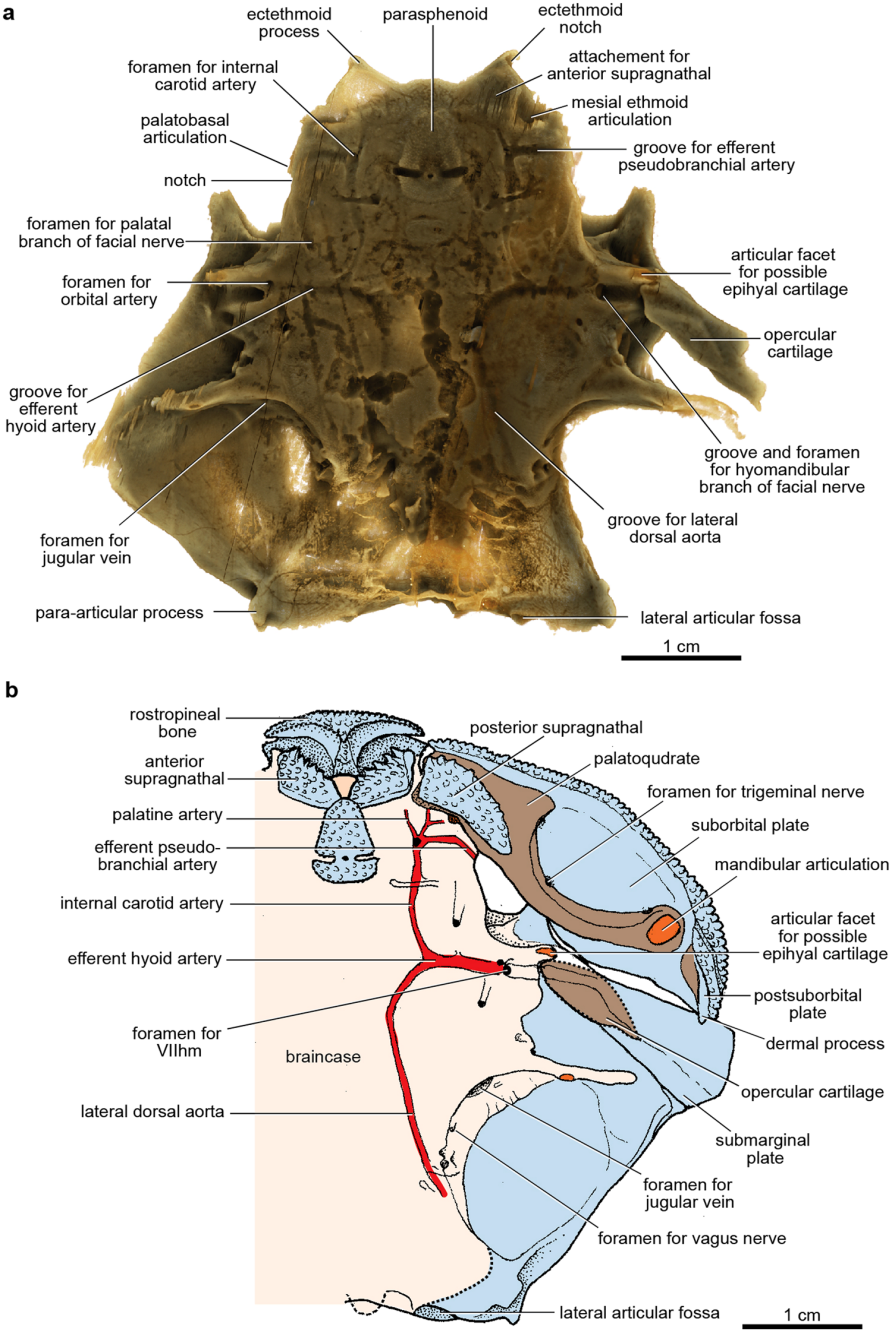
Braincase of articulated buchanosteid arthrodire (ANU V244) based on high-resolution CT. (**a**) Ventral view of braincase with dermal bones attached as preserved. (**b**) Reconstruction, based on 3D printouts, showing left side of skull and braincase in ventral view, with left cheek and operculum and upper gnathal elements in position. Light blue, dermal bones; brown, visceral arch cartilages; pale pink, braincase; orange, articular facets; red, blood vessels.

The single palatobasal articulation on the palatoquadrate (Fig. 3a) connects with a corresponding facet on the edge of the subnasal shelf, in front of a distinct notch at the lateral end of the ventral groove for the efferent pseudobranchial artery (Figs 5a and 6). This notch forms the anterior corner of a large space between the palatoquadrate and braincase when the two are articulated together (Figs 2a and 6b; Supplementary Fig. 2d). Possibly this space contained a ligamentous attachment to the braincase. Another notch farther forward, clearly seen on both sides to form a foramen between the palatoquadrate and braincase (Figs 2b and 6b; Supplementary Fig. 2d), separates the palatobasal from the ethmoid connection, and also transmitted an arterial branch, based on grooves on the ventral braincase surface (more detail is provided in Supplementary Information). The arterial system in ANU V244 is much more complex than the pattern recently represented for ‘*Buchanosteus*’ as a primitive gnathostome^7^.

With the upper gnathal bones positioned on their clearly defined attachment surfaces, a gap remains between them (Fig. 2b); presumably this permitted some flexibility of the upper jaw against the braincase. A notch between the posterior supragnathal and the palatoquadrate (Supplementary Fig. 3c) shows that the anterior groove on the palatoquadrate (Fig. 3c) probably carried a blood vessel to the ethmoid articulation. A distinct groove behind the ectethmoid process inside the anterior braincase margin (Fig. 6a) forms a corresponding lateral notch when the anterior supragnathal is placed on its attachment surface (Supplementary Fig. 3d). From its position, this notch probably carried a continuation of the same vessel. No equivalent notch or groove was identified in *Dicksonosteus* ^27^.

On all three dermal gnathal elements the lateral denticle rows are the largest. The gnathal bones are displaced in the original specimen (Supplementary Fig. 2c), but jaw reassembly using 3D printouts (Fig. 2d) shows the two main longitudinal denticle rows on the infragnathal occluded only with the larger lateral denticles of the posterior supragnathal, and along the anterior margin of the anterior supragnathal. By manipulating all jaw elements in their correct positions, a symphysial gap remains between the left and right Meckel’s cartilage (Fig. 2a). Thus, the unfinished anterior end of the cartilage must have been extended as a cartilaginous (or ligamentous) connection to form the mandibular symphysis.

### Dermal operculum and hyoid arch structures

The right postmarginal and submarginal plates make a good fit against the skull 3D printout (Fig. 2c). The submarginal plate broadens posteriorly, and differs in shape to that restored for *Parabuchanosteus*, which is more slender, with a posterior notch for the postmarginal corner of the skull roof^35, 36^. In ANU V244 the dorsal edge of the submarginal plate shows how it connected to the skull, its broader anterior part abutting inside the ventral edge of the skull roof, and the narrow posterior edge sliding against the postmarginal plate, and projecting back behind the postmarginal corner of the skull (Figs 1a and 2c; Supplementary Fig. 2d).

A perichondrally-ossified opercular cartilage is attached inside the proximal part of the submarginal plate (Figs 5b,c and 6). It has the same structure as in the arthrodires *Arctolepis* and *Dicksonosteus* ^27, 31, 37^, and some other placoderm groups, being confined to the anterodorsal corner of the dermal operculum, with no connection to any other visceral arch element^16^. In all well-preserved examples of the placoderm opercular cartilage, including our specimen, the distal end is completely enclosed by perichondral bone. Nevertheless, that it represents a much reduced epihyal element remains a prevalent interpretation, and its articulation with the braincase has been homologised with the hyomandibular articulation in other forms^7, 9^.

In ANU V244, the left submarginal is in articulated position (Fig. 6a; Supplementary Fig. 2b), and for the first time in a placoderm demonstrates the groove for the hyomandibular branch of the facial nerve passing directly from its foramen onto the opercular cartilage. It crosses to its anterior edge, where lateralis fibres of the hyomandibular nerve probably branched off to enter the adjacent mandibularis externis canal on the palatoquadrate (Figs 3b and 5b,c). The proximal end of the operculum in ANU V244 does not contact the terminal articulat facet on the anterior postorbital process, which must have been for another element (see Supplementary Information).

## Discussion

The palatoquadrate of *Entelognathus* ^2^ is interpreted to have a mesial osteichthyan-like commissural lamina enclosing the adductor fossa, but which extends forward as a ‘tunnel-like anterior extension that traverses the palatoquadrate and emerges on its mesial face’^2^. This condition is unknown in osteichthyans, but was said to be similar to many arthrodires, including *Buchanosteus*. However, our new evidence indicates that this is not the condition in basal arthrodires. As described above, all canals that communicate with the adductor fossa are situated lateral to the cartilage, between it and the dermal bone, and thus do not traverse the palatoquadrate. We suggest this was the primitive condition in placoderms, and indicates the condition for basal gnathostomes.

An assumed convex articular surface for the mandibular joint on the quadrate for ANU V244 would have articulated with the corresponding concavity on Meckel’s cartilage. In contrast, the mandibular joint in *Entelognathus* is described as a prearticular process and quadrate concavity, like acanthodians and chondrichthyans, and unlike the bipartite convex articulation of osteichthyans^2^. Our new evidence indicates that the early arthrodire condition resembled that of osteichthyans.

The dermal cheek unit comprises a large suborbital plate anteriorly and a smaller postsuborbital plate posteriorly in most arthrodires (Supplementary Fig. 4). However, in the ‘maxillate’ placoderms, additional bones equivalent to the maxilla and premaxilla of osteichthyans have been identified^2, 3^. The main cheek bone (suborbital plate) has been homologised with the jugal of osteichthyans, and the postsuborbital has been compared with the osteichthyan quadratojugal. Some insights on these homologies are provided by a consideration of the cartilage attachments inside the dermal bones of the cheek.

Typical arthrodires like *Eastmanosteus* (Supplementary Fig. 4a,d) have the autopalatine and metapterygoid parts of the palatoquadrate attached inside the dermal suborbital plate, whilst the quadrate portion is fused to the inside of the postsuborbital plate^21, 38, 39^. However, our new evidence of the buchanosteid palatoquadrate shows the mandibular joint (signifying the quadrate part of the palatoquadrate) located inside the suborbital plate, with another separately ossified cartilage, compared above with the osteichthyan interhyal, situated inside the postsuborbital plate (Supplementary Fig. 4b). This evidence raises questions about the homology of a small posterior bone in the cheek complex in *Entelognathus*, suggested to be equivalent both to the placoderm postsuborbital, and the quadratojugal of osteichthyans, on the assumption that these bones sat outside the mandibular joint^2^.

Further investigation is needed, as this small posterior dermal bone is not identified in the dermal cheek of the second maxillate placoderm *Qilinyu* ^3^. Also, the restoration of *Dicksonosteus* ^31^ suggests an intermediate condition, with the quadrate articulation straddling the suborbital-postsuborbital dermal bone boundary (Supplementary Fig. 6b). An anterior shift in the position of the *Dicksonosteus* quadrate was argued as necessary to achieve the condition in phyllolepid placoderms^40^, but they used the hypophysial foramen as a landmark, and assumed that a palatobasal connection was unknown in placoderms. However, this connection, described above, is present in several groups^16^, even if more anteriorly placed than in osteichthyans.

An incomplete postsuborbital plate with part of the ‘quadrate’ attached was identified for *Dicksonosteus*, suggesting that the quadrate was separately ossified, as previously assumed for *Romundina* ^27^. As preserved, that specimen shows no distinguishing features of the quadrate ossification, and we suggest this could possibly be a separately ossified cartilage behind the palatoquadrate with the mandibular joint confined to the suborbital plate, as in ANU V244. We consider that the cheek unit in *Dicksonosteus* and other basal arthrodires, even if carefully described previously, could be reinvestigated in the light of the new evidence provided here.

The position of this separate cartilage (Supplementary Fig. 4b) suggests comparison with the interhyal of osteichthyans, a separate element of the hyoid arch immediately behind the palatoquadrate, invariably linking it with the hyomandibula and ceratohyal. The interhyal was proposed as an osteichthyan synapomorphy^41^. Recent analyses^2, 3, 25^ have assumed the interhyal was absent in placoderms, based on the hyoid arch of ptyctodontids, where a possible interhyal^42^ has been re-interpreted as a ceratohyal^43, 44^. This character needs re-investigation for other placoderms, and the new evidence of ANU V244 demonstrates that there was an accessory element of the hyoid arch in basal arthrodires, which we propose as equivalent to the interhyal of osteichthyans.

Some ideas on recently suggested homologies between the gnathal elements of placoderms and the marginal tooth-bearing bones of osteichthyans arise from a consideration of the ethmoid connection between the palatoquadrate and the braincase. ANU V244 demonstrates there are two ethmoid articulations. The mesial articulation, located on the braincase floor just behind the attachment area for the anterior supragnathal (Fig. 6b), is comparable to the position of the ethmoid articulation in various osteichthyans, located posterolateral to the vomer (e.g. *Youngolepis* ^45^). Such similarities formed the basis for proposing homology between the anterior supragnathal and the osteichthyan vomer^34^. However, the evidence of the ‘maxillate’ placoderm *Qilinyu* has suggested an alternative homology, to the osteichthyan premaxilla^3^.

The dermal submarginal plate of placoderms is clearly the functional equivalent of the opercular dermal bones of osteichthyans, because various Early Devonian forms (e.g. *Bryantolepis*, *Dicksonosteus*, *Romundina*, *Wuttagoonaspis*) show that it was only loosely attached to the skull, as a movable operculum over the branchial region^16, 27, 46^. Within arthrodires the operculum undergoes major transformation, from a more ovate bone positioned behind the cheek in most Early Devonian forms, in Late Devonian eubrachythoracid arthrodires becoming an elongate element attached above and suspending the suborbital complex to take over the opercular function^1^.

In *Holonema*, a perichondrally ossified structure attached inside the ovate submarginal plate was interpreted as an ‘opercular cartilage’, whilst that inside the elongate submarginal in *Torosteus* was interpreted as an elongate cartilaginous epihyal or hyomandibula^21, 30^ (but we consider the opercular cartilage of *Torosteus* is incomplete; see Supplementary Information). A similar interpretation was proposed to restore an elongate epihyal in ptyctodontid placoderms from Gogo^42^, but the Gogo ptyctodontid *Materpiscis* now shows that interpretation also to be unreliable. *Materpiscis* has two separate perichondrally-ossified cartilages in this region: a small opercular cartilage inside the proximal end of the submarginal plate, and a vertically oriented epihyal, with which the opercular cartilage was evidently in articulation^42^. This supports the view that the opercular cartilage of placoderms is not homologous to the epihyal^16^.

The alternative interpretation^30, 40^, that the placoderm opercular cartilage is a reduced epihyal, forms the basis for homology with the hyomandibular articulation of osteichthyans^7, 9^. *Entelognathus*, which combines various osteichthyan and placoderm features, has a partially exposed elongate crescentic dermal ‘opercular’ bone homologised with the placoderm submarginal plate, also with a small internal opercular cartilage^2^, essentially as described above. Our new evidence demonstrates two separate articular areas on the anterior postorbital process in buchanosteids. The position of the opercular cartilage connection is very similar to that restored for *Dicksonosteus* ^27, 31^. The extra articulation (Supplementary Fig. 2b), previously identified as for the opercular cartilage (Supplementary Fig. 6a), must be for an additional element, because an otic connection with the palatoquadrate, as interpreted for *Dicksonosteus* ^31^ and *Romundina* ^4^, is not possible when 3D printouts are reassembled (Fig. 5b,c). A clear gap between the palatoquadrate and this articulation could have accommodated an additional unossified epihyal element (Fig. 5c), contrary to the previous claim that ‘there is no room to insert a further element’ in that position^30^. The 3D printouts also show that the extra dorsal articulation on Meckel’s cartilage could have contacted this unossified element (Fig. 5b,c).

The new evidence from this articulated placoderm (ANU V244) reveals much more complexity regarding composition and articulation of the hyoid arch, shown to be key characters in resolving placoderms as either monophyletic or paraphyletic^9^. For the first time, we establish jaw morphology and functional connections of the jaw cartilages to the braincase, using the evidence of the position of major cranial nerves and vessels to confirm homologies with other groups. Such information can only be ascertained from this unique articulated arthodire, making it a key taxon for resolving basal gnathostome relationships.

## Material and Methods

Scanning was done on two machines developed and built in the Department of Applied Mathematics, ANU^47, 48^. The scanning data was 3D rendered and segmented using the programs *Drishti 2*.*5* and *DrishtiPaint* (http://nci.org.au/nci-systems/scientific-visualisation/visualisation-services/) and separate components of the head skeleton were printed at 6 times natural size on a ZPrinter 650. More detail is given in Supplementary Information.

The 3D format files of the infragathal, Meckel’s cartilage and suborbital, and a movie showing the posterior supragnathal, infragathal and suborbital plate in position are available on NCI (National Computational Infrastructure, Australia) Catalo﻿gue: http://dx.doi.org/10.4225/41/597﻿8116a9e671.

## Electronic supplementary material

Supplementary Information
