# Supplementary material for: New findings in a 400 million-year-old Devonian placoderm shed light on jaw structure and function in basal gnathostomes
Source: Sci Rep. 2017 Aug 10;7:7813. doi: 10.1038/s41598-017-07674-y (PMC5552730; doi:10.1038/s41598-017-07674-y)
Supplement: Supplementary file 1 — Supplementary Information [file 41598_2017_7674_MOESM1_ESM.pdf]

Supplementary materials for

New findings in a 400 million-year-old Devonian placoderm shed light on jaw structure and function in basal gnathostomes

Yuzhi Hu[1,2,+], Jing Lu[1,3,+] and Gavin C. Young[1,*]

[1] *Department of Applied Mathematics, Research School of Physics and Engineering, Oliphant Building 60, Australian National University, Canberra, ACT, Australia 2601*

[2] *Research School of Earth Sciences, Building 142 Mills Road, Australian National University, Canberra, ACT, Australia 2601*

[3] *Key Laboratory of Vertebrate Evolution and Human Origins of Chinese Academy of Sciences, Institute of Vertebrate Paleontology and Paleoanthropology, Chinese Academy of Sciences, Beijing, China 100044*

[*] *To whom correspondence should be addressed: gavin.young@anu.edu.au*

[+] *these authors contributed equally to this work*

This supplementary Information includes:

Supplementary Notes

Supplementary Figures

Supplementary References

Supplementary Notes

Geological provenance, specimen locality data, and repository

The largest exposure of Early Devonian limestones in southern New South Wales occurs in two separate outcrops around Lake Burrinjuck, a man-made reservoir on the Murrumbidgee River about 50 km NW of Canberra (Supplementary Fig. 1a). Specimen ANU V244 was found by Mr Ian Thompson in 1977, preserved in a small limestone nodule. The fossil locality is near 'Shearsby's Wallpaper', a reserved heritage fossil site in the eastern limestone outcrop ~2.5 km downstream from Taemas Bridge (Supplementary Fig. 1b). The stratigraphic level is within the '*Spirifer' yassensis* Member of the Taemas Formation (Supplementary Fig. 1c). Conodont dating shows the Pragian-Emsian boundary occurs within the underlying Cavan Limestone, and the upper limestones of the Taemas Formation are within the Emsian *serotinus* zone[1], so the fossil horizon lies within the Emsian stage of the Early Devonian. The Shearsby's Wallpaper site has produced other significant specimens, including the second skull of the lungfish *Dipnorhynchus*[2,3], as well as the only previously described example of the buchanosteid palatoquadrate[4].

With a vertebrate faunal list of some 62 genera and 70 species, the Burrinjuck fossil fish assemblage is the most diverse fossil fish fauna known from the Devonian, and also the oldest coral reef fish assemblage from the fossil record[1].

Institutional abbreviations

Institutional abbreviations for registered specimens are: ANU, College of Science, The Australian National University, Canberra, ACT; WAM, Western Australian Museum, Perth, WA.

Taxonomy

ANU V244 was previously referred to as a 'new buchanosteid'[6], a member of a complex of small to medium-sized arthrodires from the Burrinjuck fossil fish locality[7]. The genus *Buchanosteus* was erected[8] for a skull specimen from Early Devonian limestones of Buchan, Victoria, first named as '*Coccosteus osseus*'[9]. New species and genera have been documented from the Early Devonian limestones of southeastern Australia[7,10,11], and isolated skulls and other bones are known from rocks of similar age in Russia, Saudi Arabia, and China[12-14]. The superfamily Buchanosteoidea [15] was recently redefined[7] to contain two families (Buchanosteidae, Parabuchanosteidae), with two new buchanosteid species and genera: *Richardosteus barwickorum* and *Urvaspis lithuanica*. The specimen used in this study (ANU V244) belongs to the Buchanosteoidea, but its precise species and relation to other described taxa is not yet established. It displays numerous morphological features unknown in other fossils, and is the only known specimen showing the dermal bones of the cheek associated with the skull roof and braincase (Fig. 1). However the median dorsal and anterior dorsolateral plates of the trunk-armour are missing, and the overlaps on the latter were used to define the family Parabuchanosteidae[7].

We note that the parasphenoid of ANU V244 has the same shape as in *Buchanosteus confertituberculatus*, with transverse grooves that are widely open laterally (Supplementary Fig. 5) as diagnosed for the family Buchanosteidae[7]. In addition, ANU V244 lacks a subpituitary fenestra, like *Buchanosteus confertituberculatus*[7,9], whereas this opening is well developed in *Parabuchanosteus*[4,10]. Furthermore, the dermal opercular bone (submarginal plate) has a different shape to the isolated bone that was referred to *Parabuchanosteus*[10], and the postmarginal plate forming the lateral corner of the skull is a small triangular bone, in this respect resembling *Buchanosteus* rather than *Parabuchanosteus*[7]. However, the ridges on the lateral trunk-armour plates are absent in the trunk-armour specimen referred to *Buchanosteus confertituberculatus*[7]. For these reasons we consider that ANU V244 is probably a new taxon within the family Buchanosteidae, and it is referred to in the text as a 'buchanosteid'.

The genus *Buchanosteus* has been scored as a basal arthrodire in data matrices supporting various gnathostome phylogenies, but the described morphology[4] derives from a range of incomplete specimens including some earlier described as *Parabuchanosteus*[10], on the assumption there was only one genus (*Buchanosteus*) in one family[4]. However, with at least three genera in two families now recognised from SE Australia[7], and many new specimens indicating greater diversity, the morphology used in phylogenetic analyses is considered a composite of closely related forms, so we have referred to the taxon as '*Buchanosteus*'.

Specimen preparation

ANU V244 was partly acid-etched from the limestone matrix using formic acid by G.C.Y. in Paris in 1999 (laboratory of the Muséum national d'Histoire naturelle). In this process some of the jaw elements were detached and studied in detail[6]. However, the major morphological features in the inner parts of this fragile and unique specimen were not accessible until new techniques of micro-CT scanning became available.

Micro-CT scanning

ANU V244 was scanned in 10/9/2011 and 15/4/2015 on instruments developed and built at the ANU Department of Applied Mathematics. The 2011 XCT scan used an instrument with a 80kV/110uA reflection-style X-ray source and 2 mm thick silicon dioxide filter. The specimen was placed 72 mm from the source, and the detector positioned 350 mm from the source. The specimen was imaged through 360 degrees rotation. Reconstruction was based on 2880 radiographic projections formed on a 2048 × 2048 Perkin Elmer Flat Panel camera. The 2015 rescan used a new double-helix HeliScan CT Scanner with higher resolution and a new algorithm to process and reconstruct CT data. 1.2 mm aluminium and 0.35 mm stainless steel filters were used, with specimen distance 85 mm from the source, and detector position 396 mm from the source, and probed separately with a polychromatic X-ray beam (Bremsstrahlung radiation). Accelerating voltage of the electron beam generating the Bremsstrahlung radiation was 110kV with a current of 100μA. A series of X ray transmission

radiographs, collectively called the projection data, were acquired by the detector as the specimen was rotated through 360 degrees double-helically over a period of 18 hours. Reconstruction was based on 2520 radiographic projections formed on a 2048 × 1538 Varian Flat Panel camera.

Visualization, segmentation, 3D rendering

XCT scanning data was processed using the program *Drishti 2.5* (see http://nci.org.au/nci-systems/scientific-visualisation/visualisation-services/) developed to visualize 3D tomography and electron-microscopy data. Segmentation of the separate components (rostral capsule, postethmoid skull and braincase, optic capsules, jaw and cheek elements, toothplates) was undertaken using *DrishtiPaint* on 1589 slices from the 2011 scan. The 'suborbital complex' (dermal cheek unit with attached perichondrally ossified upper jaw cartilages) was also re-segmented from the 2015 data set.

The final output was sent to a ZPrinter 650 in Stanford Triangle Format (. ply). Additive manufacturing technology creates a printout by successive layering of cross-sectional slices from the tomographic data (layer thickness 0.1 mm). The printouts are built from powdered gypsum and then strengthened with cyanoacrylate resin. Elements were printed separately, enlarged by a factor of 6 for detailed investigation of the morphological fit between the various skull and jaw components. Of the paired upper gnathal elements, only one anterior supragnathal was preserved, so an opposite element was produced by reversing the CT data. The other gnathal elements (posterior supragnathal, infragnathal), and the jaw cartilages, all showed essentially identical morphology between left and right sides.

Additional notes on morphology

Supplementary Figs 2-4 illustrate more detail on reassembly of jaw elements, their attachments to the braincase, and relationship to the opercular unit (submarginal plate plus opercular cartilage).

The incomplete left submarginal plate and opercular cartilage are preserved with the proximal end still in articulation against the anterior postorbital process of the braincase (Supplementary Fig. 2b). For the first time in a placoderm, this demonstrates the groove for the hyomandibular branch of the facial nerve passing directly from its foramen onto the opercular cartilage, the groove crossing to its anterior edge. From this point, lateralis fibres of the hyomandibular nerve probably branched off to enter the adjacent mandibularis externis canal on the palatoquadrate. Distally the groove deflects back to the posterior edge of the cartilage (see also Figs. 5b, c; 6a). The efferent hyoid artery passed laterally from the anterior postorbital process[4,16], and is also represented by a strong groove in ANU V244 (Fig. 6; Supplementary Fig. 5). This artery may have also occupied the grooves shown on the opercular cartilage. By comparison, in early osteichthyans (e.g. *Eusthenopteron*)[17] two nerves (r. mandibularis VII, r. mandibularis lateralis) and a hyoid vein are also restored to pass down the hyoid arch.

The opercular cartilage in ANU V244 is forked to enclose the base of the anterior postorbital process, the dermal bone wrapping around it anteriorly, and the cartilage posteriorly (Supplementary Fig. 2b). Neither is in contact with the extra (terminal) articular facet on the end of the anterior postorbital process, previously interpreted for '*Buchanosteus*' as either for the opercular cartilage[4] (Supplementary Fig. 6a), or called the hyomandibular articulation[18]. This new evidence demonstrates two separate articular surfaces on the anterior postorbital process, a condition previously recognized in only a few other arthrodires[19,20]. In those instances the terminal articular surface was assumed to contact the palatoquadrate in an 'otic' connection, as restored for *Dicksonosteus* (Supplementary Fig. 6b).

In ANU V244 this terminal articulation is a flat articular facet, elongated in the direction of the long axis of the opercular cartilage, but facing ventrolaterally, so contact with the opercular cartilage was impossible (Supplementary Fig. 2b). Although close together, any consideration that the two articulations could have received a single element like the 'double-headed' hyomandibula of sarcopterygians (e.g. *Eusthenopteron*[17]) can be discounted. However, this is a possible interpretation for *Romundina*, where adjacent articular areas on the posterior face of the anterior postorbital process have been attributed to hyoid arch elements[21]. Various other placoderms show two separate articulations associated with the exit of the hyomandibular nerve from the braincase, that anterior to the nerve foramen interpreted as for the hyomandibula[22], or for an otic process of the palatoquadrate[19,21]. However, reassembly of 3D printouts for our specimen also demonstrates that the extra articulation could not have contacted the palatoquadrate (Supplementary Fig. 2d). By comparison, in the arthrodire *Dicksonosteus*, and in some *Kujdanowiaspis* specimens[16], there is only a single keyhole-like articulation on the posterior side of the anterior postorbital process. This corresponds precisely to the opercular cartilage articulation as preserved on the right side of ANU V244 (Supplementary Fig. 5).

The opercular cartilage in ANU V244 is clearly a closed structure, in contrast to the description of the Gogo arthrodire *Torosteus*, where this element was interpreted as open-ended, with an unossified continuation to the distal end of the submarginal plate. On that basis it was interpreted as an epihyal rather than an opercular cartilage[23]. However we consider the structure in *Torosteus* is incomplete distally, with a broken end (G.C.Y., personal observation of WAM 88.2.7). We suggest therefore that the opercular cartilage in *Torosteus* was also completely closed by perichondral bone, as demonstrated in our specimen, and other Early Devonian arthrodires (e.g. *Arctolepis*, *Dicksonosteus*), precluding any distal cartilaginous extension.

The clarification of the mandibular joint position (Supplementary Fig. 3a, b) is based on fitting the dermal gnathal elements to the palatoquadrate and Meckel's cartilage, and then placing them in occlusion. The dermal cheek complex of the Gogo arthrodire *Eastmanosteus*[24] is used to exemplify the typical position of the quadrate ossification on the postsuborbital plate (Supplementary Fig. 4a, d). On the assumption that this was a general placoderm condition, the perichondral ossification inside the suborbital plate of *Romundina* was interpreted to comprise only the autopalatine and

metapterygoid, and the quadrate was assumed to be separately ossified, and fused to the inside of the postsuborbital plate (both elements unknown)[22,25]. We present a re-interpretation of the *Romundina* palatoquadrate (Supplementary Fig. 4e) to revise the previous interpretation[22], on the assumption of similar morphology to that revealed by ANU V244, with the palatoquadrate restricted to the dermal suborbital plate.

Romundina displays the primitive 'omega' shape for the placoderm palatoquadrate[26], which was based on the Gogo arthrodire *Holenema*, at that time the only one showing a completely ossified palatoquadrate[27]. This was contrasted with a generally 'cleaver-shaped' palatoquadrate in various other groups (acanthodians, chondrichthyans, osteichthyans). Buchanosteids have larger orbits than *Holenema*, and the orbits are more laterally placed that in *Romundina* or *Bothriolepis*, giving the cheek a distinct suborbital section (equivalent to the 'handle' of a cleaver) similar to the condition in crown gnathostomes. The major difference to crown gnathostomes is the posterior embayment for the adductor fossa, in the buchanosteid palatoquadrate. This cannot be explained only by the muscle space being confined by the dermal cheek bones, because early osteichthyans (also with a dermally enclosed cheek) have a continuous 'commissural lamina' between autopalatine and quadrate parts of the palatoquadrate. This encloses the adductor fossa mesially[17], to provide a cleaver-like shape.

Originally, four articulations were inferred from the single incompletely preserved acid-etched palatoquadrate of *Romundina*[22]. Two of these represented an ethmoid ('orbital') connection as identified on the braincase[28], but better detail now indicates three articulations for the autopalatine in *Romundina*[21]. The posterior one was provisionally interpreted as part of the palatobasal connection by comparison with '*Buchanosteus*'[22]. The anterior articulation is located ventrally and mesially, and clearly corresponds to the mesial ethmoid articulation described for ANU V244, as it would also be located posterolateral to paired anterior supragnathals – these are preserved in a closely related acanthothoracid (specimen CPW.9)[28]. It is noted that the infolded ventral tuberculate surface in this specimen could have formed the biting margin of the mouth (a posterior supragnathal is not known in *Romundina*), and this structure may be more prominently developed in other acanthothoracids[29]. However whether it could have formed the biting margin depends on the orientation of the suborbital plate against the skull. In our buchanosteid this proved to be very oblique in order to place the gnathal elements in occlusion. Again, such detail can only be established by using micro-CT scanning and 3D printing techniques on rare articulated specimens, as demonstrated above.

The Meckel's cartilage was noted above to be deepest towards the anterior end, and using 3D printouts to place it in occlusion with the palatoquadrate shows this corresponds to the anterior extent of the adductor fossa (Supplementary Fig. 2e). In contrast, in Palaeozoic elasmobranchs Meckel's cartilage tends to be deepest posteriorly[30], but the adductor fossa on the palatoquadrate is entirely postorbital, indicating that the muscle was more posteriorly placed. Regarding the mandibular joint, the dorsal articulation on the buchanosteid Meckel's cartilage has a corresponding position to the 'mandibular knob' in *Gogoselachus*[31]. The mandibular

knob in various Palaeozoic and modern elasmobranchs forms a secondary articulation with a concavity on the palatoquadrate. but this was clearly not so in the buchanosteid jaw, where the dorsal articulation does not contact the palatoquadrate (Supplementary Fig. 3b).

Supplementary Fig. 5 gives additional information on the cranial arteries and veins as indicated by grooves and foramina on the ventral surface of the braincase. These are more completely preserved, and show much more detail, than the various specimens used for the previous restoration of the ventral braincase surface of '*Buchanosteus*'[4] (Supplementary Fig. 6a). A full interpretation will depend on segmentation of internal braincase structures, not yet carried out, but we note here that the grooves indicate a more complex pattern of arteries than recently represented for '*Buchanosteus*' as a primitive gnathostome[18].

The left groove for the lateral dorsal aorta is best preserved, with a similar position to the previous description for '*Buchanosteus*'[4](Supplementary Fig. 6a). However it extends farther posteriorly to the posterolateral edge of the ventral braincase surface, close to the anterior end of the row of spino-occipital nerve foramina. A curved lateral groove adjacent to the first of these may represent the occipital artery emanating from the lateral dorsal aorta. This is based on comparison with a similar foramen at the posterior end of the aortic canal in *Brindabellaspis*, previously identified as for the occipital artery[32], which forms a similar curved groove crossing the anterior spino-occipital nerve foramen.

The aortic groove in ANU V244 curves laterally towards the large foramen for the hyomandibular nerve, and presumably received the efferent hyoid artery passing up from the hyoid arch, as previously interpreted (Supplementary Fig. 6a). A distinct foramen shows where the orbital artery branched from the efferent hyoid artery to pierce the braincase floor, and emanate in the rear wall of the orbital cavity. This is essentially as previously described[4]. Not previously documented for '*Buchanosteus'* is a strong groove for the internal carotid artery, branching off the efferent hyoid groove with a shape showing clearly that blood flow direction was inward, from the efferent hyoid artery. The internal carotid groove curves forward on both sides to a distinct foramen lateral to the parasphenoid, where this major artery entered the braincase, as previously identified in other arthrodires[4,16,19].

Behind the parasphenoid the aortic groove crosses a transverse groove passing laterally from paired foramina. From their position these presumably opened dorsally into the interorbital canal for the pituitary vein, as previously described[4]. These grooves are therefore interpreted as parts of the hypophysial vein system. The preserved detail shows that the internal carotid was morphologically ventral to the vessel within the transverse groove. Fainter grooves pass anteriorly from each hypophysial vein foramen, and curve in towards the lateral notches of the parasphenoid. This course closely matches the position of the restored hypophysial vein in *Kujdanowiaspis*[19] and *Dicksonosteus*[16]. However, for '*Buchanosteus'* it was demonstrated by the acid-etched specimens that the depression in the lateral notch of the parasphenoid had no foramen for the hypophysial vein in this position[4]. Other parts of the cranial venous system are generally not visible in ventral view, the jugular

vein being completely enclosed in a canal within the lateral cranial wall. Its posterior opening is visible behind the posterior postorbital process, and an opening at the base of that process probably transmitted a posterior postorbital vein, associated with branches of the glossopharyngeal nerve, as previously interpreted[4].

At the internal carotid foramen a strong lateral arterial branch is indicated by a groove passing to the palatobasal connection for the palatoquadrate, on the edge of the subocular shelf. This contained the efferent pseudobranchial artery, which in ANU V244 passed posterolaterally into a notch behind the palatobasal articular facet (Fig. 6c). In other specimens of '*Buchanosteus*' there was a double palatobasal articulation (Supplementary Fig. 6a), with the efferent pseudobranchial artery evidently passing between them[4,22].

Not previously identified in any arthrodire is an additional arterial groove passing forward from the internal carotid foramen. This has a corresponding position to a groove in *Brindabellaspis* interpreted to have carried the palatine artery[32], by comparison with *Glyptolepis*[33], and the same groove was identified in *Romundina*[32]. Recently this groove in *Romundina* was labelled as for the internal carotid[21], but this seems erroneous. In ANU V244 the palatine groove branches anteriorly, with a main mesial branch directed to the anterior end of the parasphenoid. This gives off numerous smaller branches forming a reticulating network through the attachment area for the anterior supragnathal. This was evidently the main arterial supply to this gnathal element. A third branch in this arterial system is indicated by another groove originating from the efferent pseudobranchial artery immediately lateral to its junction with the internal carotid (Fig. 6b). This passes anterolaterally to the mesial ethmoid articulation, with a smaller branch reaching another notch on the edge of the subocular shelf, between the palatobasal and lateral ethmoid articulations for the palatoquadrate. 3D printouts, with the palatoquadrate in position against the braincase (Fig. 2b), show this notch forming a clear foramen on both sides. Given its connection with the arterial groove system, it can be assumed that the blood supply to the floor of the orbital cavity passed up through this foramen.

The same system is connected to the 'transverse groove' on the palatoquadrate attachment surface for the posterior supragnathal (Fig. 3), so presumably also carried arterial blood to this region. We note that there seems no corresponding arterial branch in the living shark *Chlamydoselachus*[34], nor in the Devonian placoderms *Kujdanowiaspis*[19], *Dicksonosteus*[16], or *Romundina*[21]. The other grooves also evidently transmitted vessels or nerves between the cartilage and the gnathal plates, because reassembly of 3D printouts shows that the inner ridge along the lateral edge of the posterior supragnathal (Fig. 1c) did not fill the anterior groove of the palatoquadrate, leaving an 'anterior notch' (Supplementary Fig. 3c). In articulation, this notch sat opposite a 'lateral notch' (Supplementary Fig. 3d), formed between the anterior supragnathal and a groove inside the ectethmoid process of the braincase (Fig. 6).

Supplementary Figures

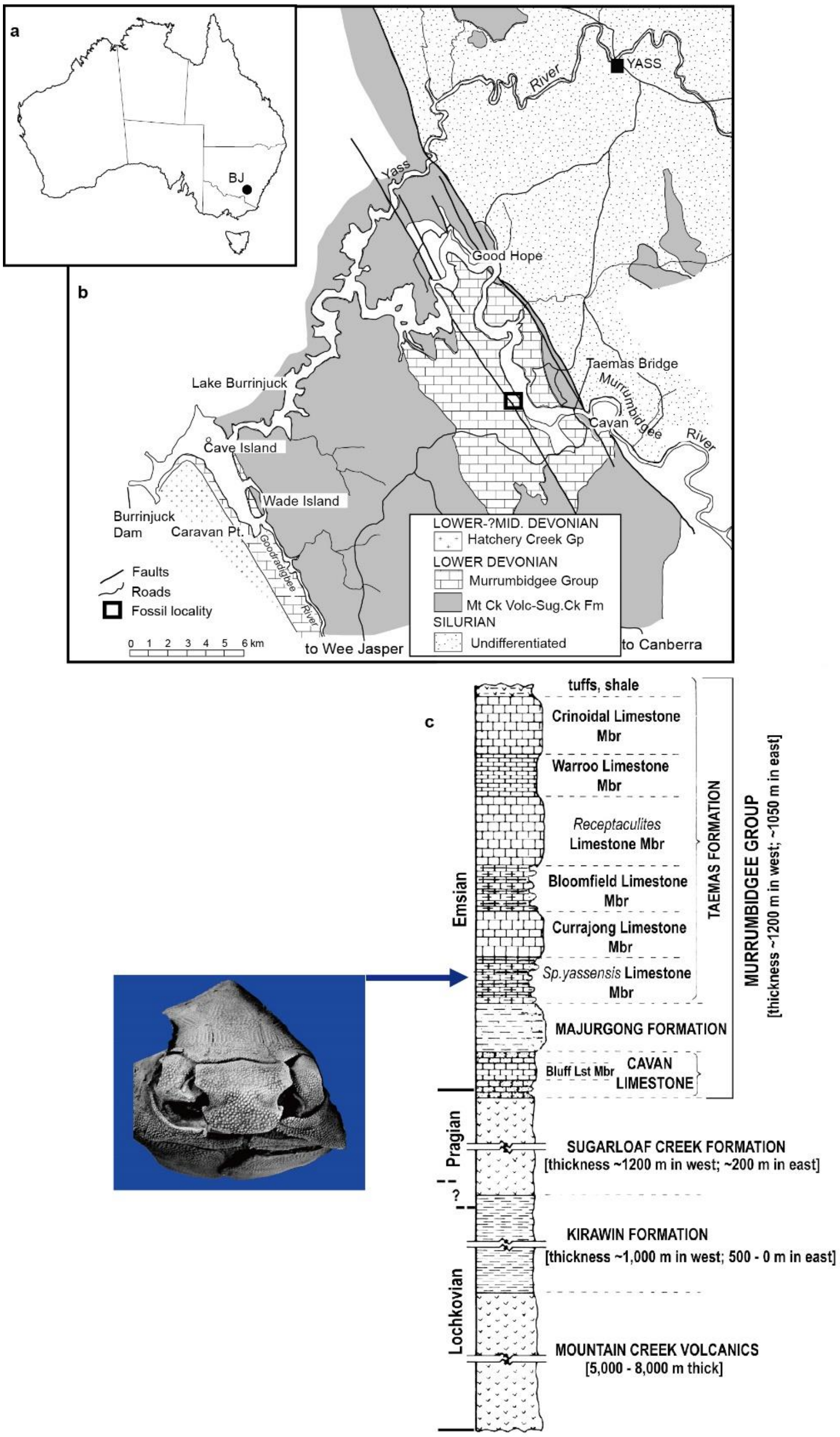

Supplementary Figure 1. Locality details for articulated buchanosteid arthrodire (ANU V244). (**a**) Location in southeastern Australia of the Early Devonian limestones at Burrinjuck (BJ), near Canberra. (**b**) Devonian geology in the vicinity of Lake Burrinjuck, showing the fossil locality for ANU V244. (**c**) Devonian stratigraphy at Burrinjuck, showing the fossil horizon for ANU V244. Maps and stratigraphic column were generated with Adobe Illustrator CS6 (http://www.adobe.com/cn/products/illustrator.html)

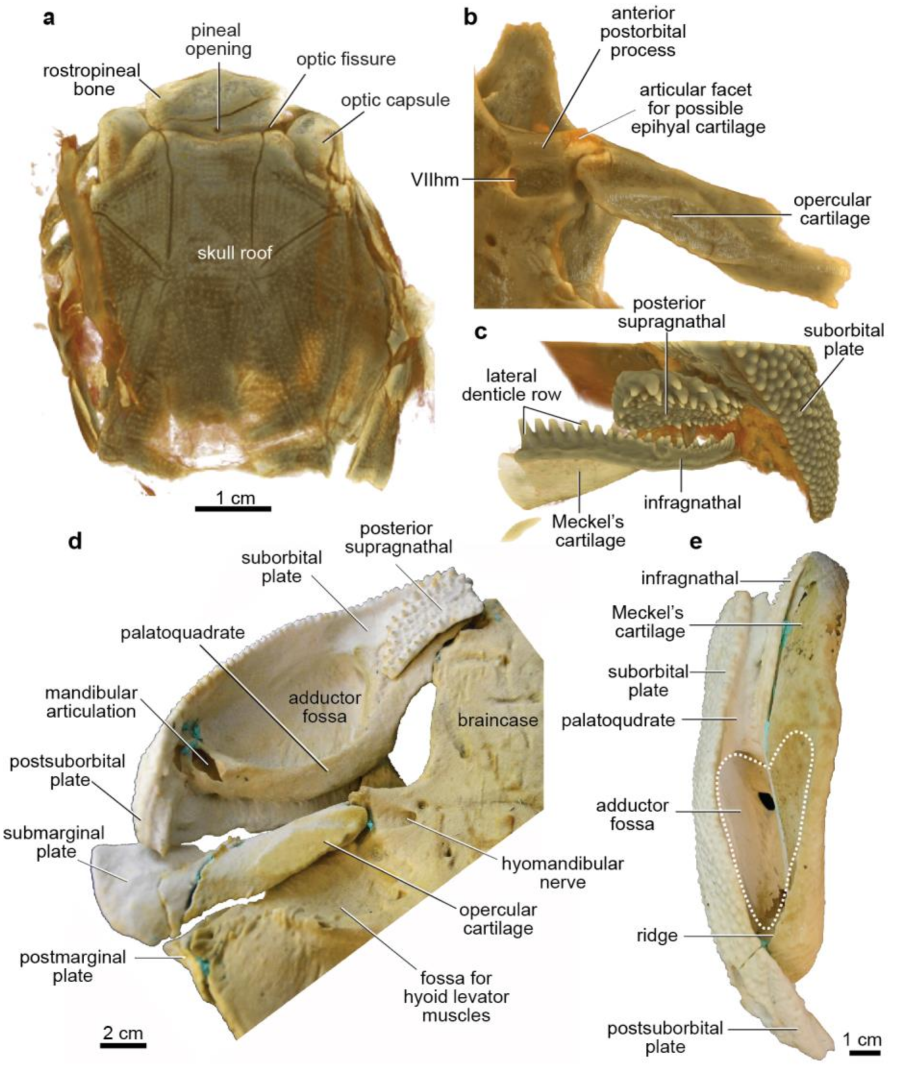

Supplementary Figure 2. Articulated buchanosteid arthrodire (ANU V244), based on high-resolution CT (a-c) and 3D printouts (d-e). (**a**) Specimen in dorsal view. (**b**) Ventral view showing detail of the left submarginal plate (incomplete) and opercular cartilage in articulation against the anterior postorbital process of the braincase, with the hyomandibular nerve foramen (VIIhm) opening in a groove crossing onto the opercular cartilage. (**c**) Preserved association of the posterior supragnathal, infragnathal and Meckel's cartilage inside the specimen. (**d**) Ventromesial view showing reassembly of the right cheek complex (dermal suborbital and postsuborbital plate, plus palatoquadrate), and right opercular unit (submarginal plate plus opercular cartilage), in articulation against the braincase. (**e**) Ventral view of the right jaw with posterior supragnathal and infragnathal in occlusion, and Meckel's cartilage in articulation on the palatoquadrate. Dotted line outlines the limits of the adductor fossa across the two cartilage elements.

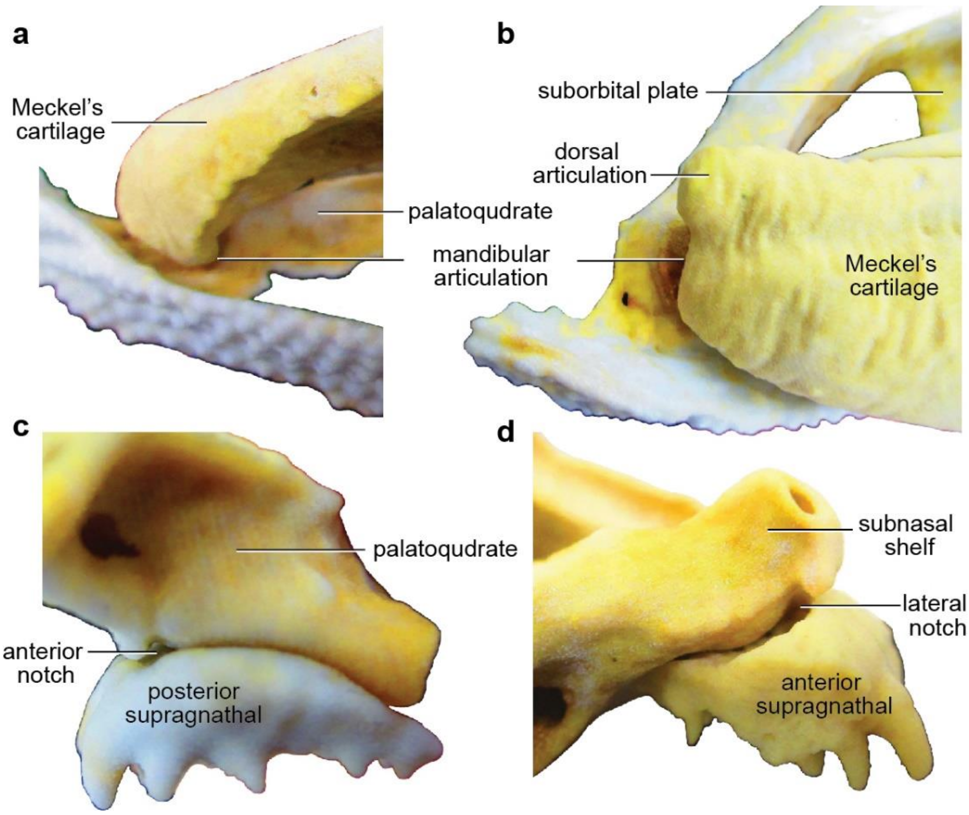

Supplementary Figure 3. Articulated buchanosteid arthrodire (ANU V244), based on 3D printouts. (a) Ventral view and (**b**) mesial view of the left mandibular joint in articulation. (**c-d**) Detail of the ethmoid connection of the palatoquadrate against the braincase, showing the articular surface at the anterior end of the right palatoquadrate, with posterior supragnathal attached (**c**), and the corresponding right lateral face of the subnasal shelf of the braincase, with anterior supragnathal attached (**d**). Not to scale.

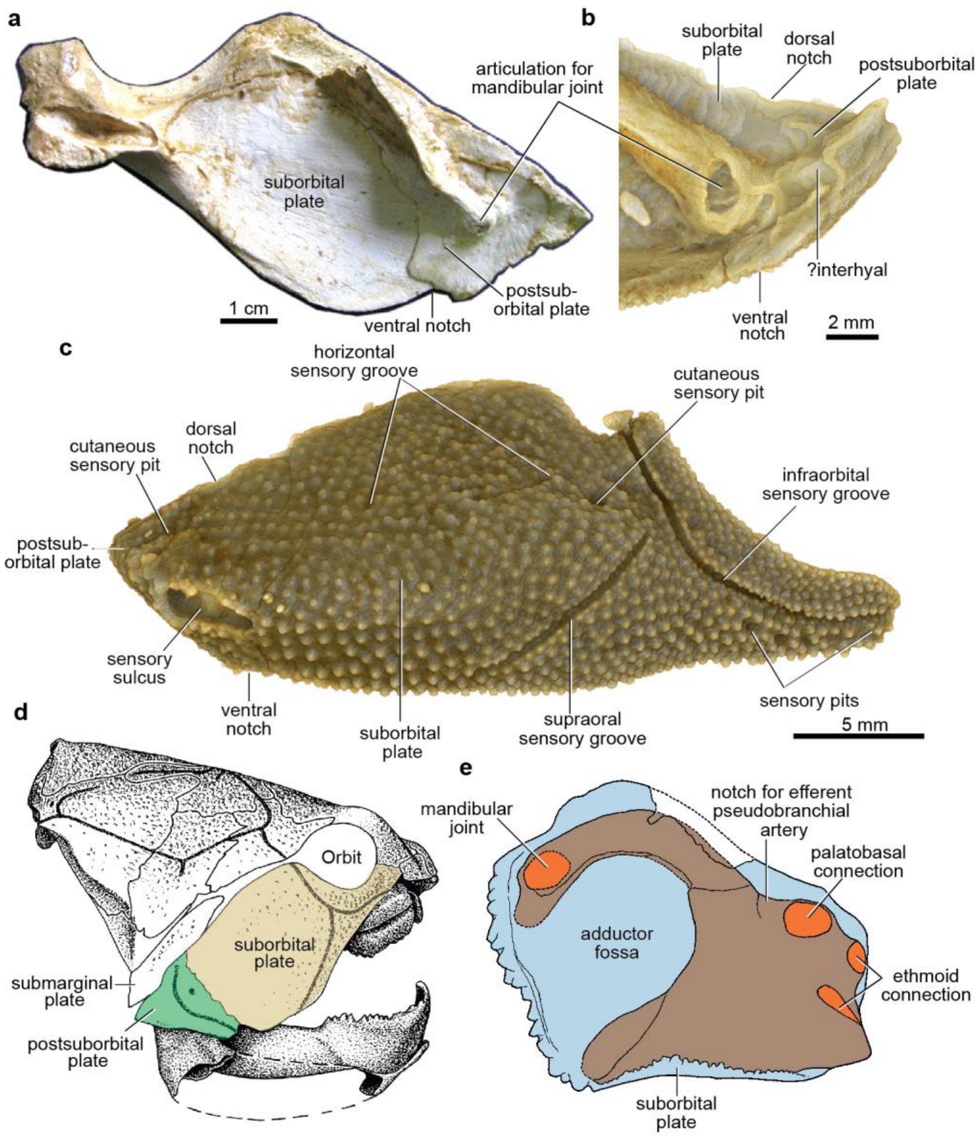

Supplementary Figure 4. Comparisons of the dermal cheek unit, attached palatoquadrate, and mandibular joint articulation in various placoderms. (**a**) Internal view of the right suborbital and postsuborbital plates with attached perichondrally ossified quadrate in *Eastmanosteus* sp. from Gogo (ANU 21633). (**b-c**) Right cheek unit of buchanosteid arthrodire ANU V244, based on high-resolution CT. (**b**) Internal view of posterior end showing the mandibular joint on the quadrate (attached to the dermal suborbital plate), behind which is a separately ossified element compared with the osteichthyan interhyal (attached to the postsuborbital plate). (**c**) External view of the right cheek unit (suborbital and postsuborbital plates). (**d**) *Eastmanosteus calliaspis* from Gogo in lateral view (modified after ref. 24), with right cheek unit (suborbital and postsuborbital plates) highlighted. **(e)** Restoration in internal view of the left suborbital plate and attached palatoquadrate of *Romundina stellina* (modified after refs. 22, 25), showing a new interpretation of the position of the mandibular joint. Light blue, dermal bones; brown, visceral arch cartilages; orange, articular facets. (**d**) and (**e**) are not to scale.

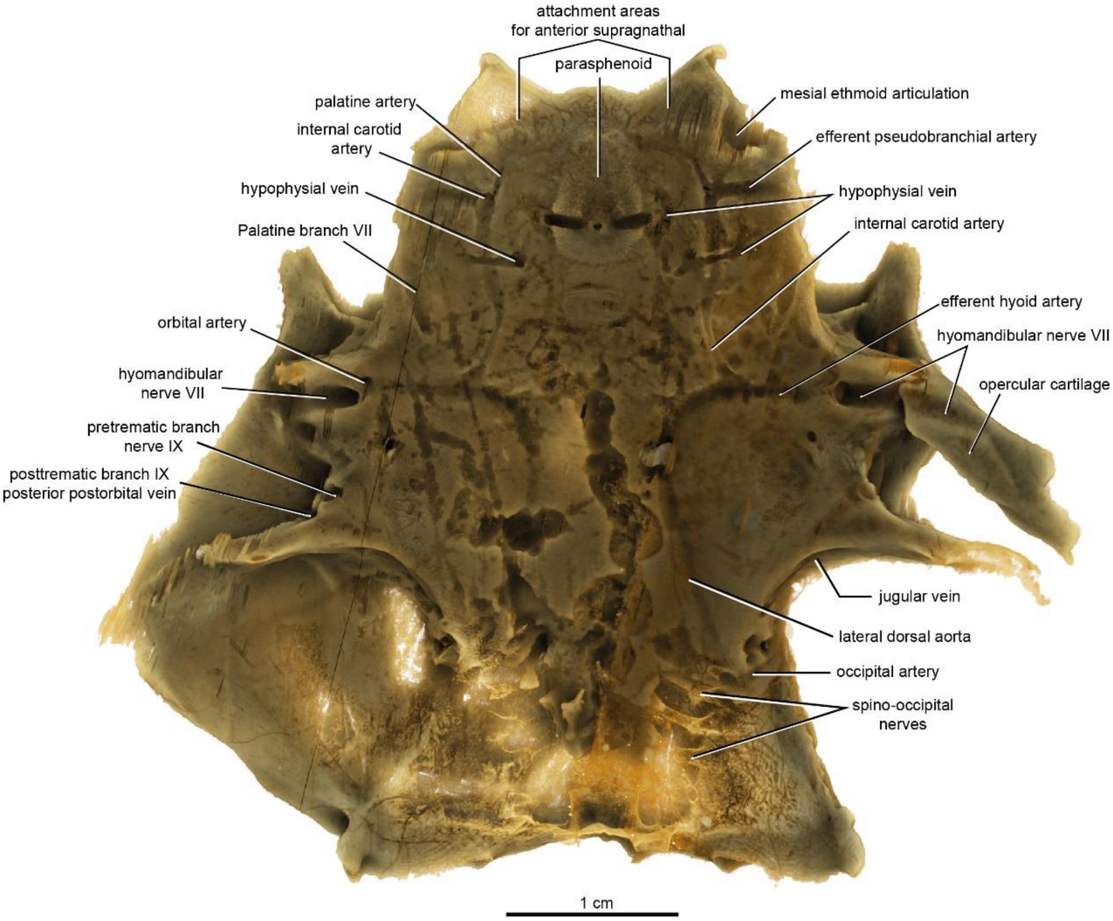

Supplementary Figure 5. Articulated buchanosteid arthrodire (ANU V244), based on high-resolution CT; interpretation of grooves on the ventral braincase surface. Interpretations are based on previous analyses, in particular for *Kujdanowiaspis*[19], ‘*Buchanosteus*’[4], and *Dicksonosteus*[16,20]. Grooves and foramina are labelled according to their contained structures.

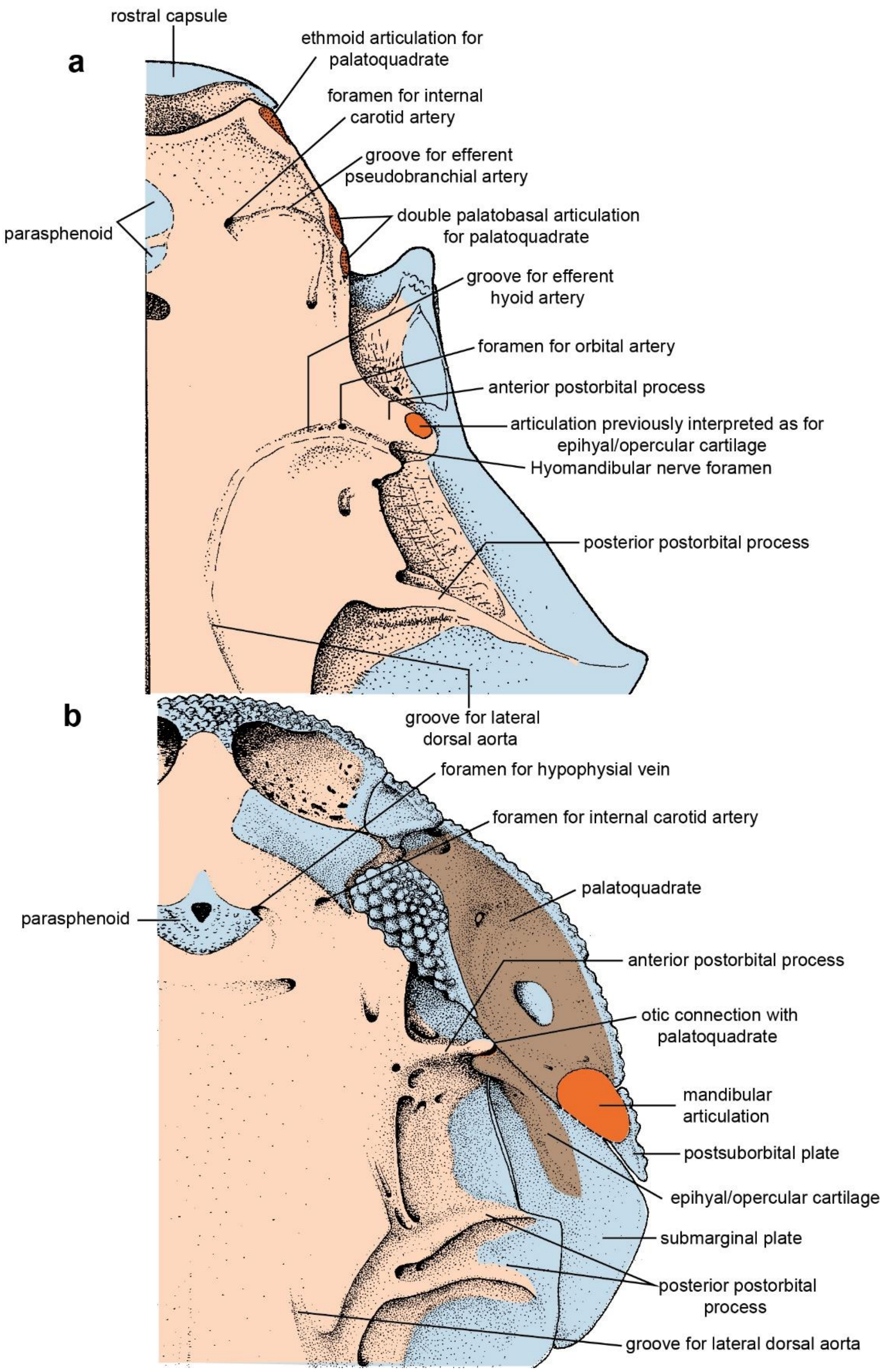

Supplementary Figure 6. Previous interpretations for '*Buchanosteus*' (a) and *Dicksonosteus* (b). (**a**) Structures on the ventral braincase surface of '*Buchanosteus*' as interpreted by ref. 4. (**b**) restoration of the ventral braincase surface, dermal cheek plates, upper jaw cartilages and gnathal elements in *Dicksonosteus*, modified after ref. 16. Light blue, dermal bones; brown, visceral arch cartilages; pale pink, braincase; orange, articular facets; red, blood vessels. Not to scale.

Supplementary References

1. Young, G. C. Wee Jasper-Lake Burrinjuck fossil fish sites: Scientific background to national heritage nomination. *Proc. Linn. Soc. N. S. W.* **132**, 83–107 (2011).
2. Thomson, K. S. & Campbell, K. S. W. The structure and relationships of the primitive Devonian lungfish - *Dipnorhynchus sussmilchi* (Etheridge). *Bull., Peabody Mus. Nat. Hist.* **38**, 1–109 (1971).
3. Campbell, K. S. W. An almost complete skull roof and palate of the Dipnoan *Dipnorhynchus sussmilchi* (Etheridge). *Palaeontology* **8**, 634–637 (1965).
4. Young, G. C. New information on the structure and relationships of *Buchanosteus* (Placodermi: Euarthrodira) from the Early Devonian of New South Wales. *Zool. J. Linn. Soc.* **66**, 309–352 (1979).
5. Hunt, J. & Young, G. C. A new placoderm fish of uncertain affinity from the Early-Middle Devonian Hatchery Creek succession at Wee Jasper, New South Wales. *Alcheringa* **35**, 1–23, doi:10.1080/03115511003793645 (2011).
6. Young, G. C., Lelièvre, H. & Goujet, D. Primitive jaw structure in an articulated brachythoracid arthrodire (placoderm fish; Early Devonian) from southeastern Australia. *J. Vertebr. Paleontol.* **21**, 670–678 (2001).
7. Long, J. A., Mark-Kurik, E. & Young, G. C. Taxonomic revision of buchanosteoid placoderms (Arthrodira) from the Early Devonian of south-eastern Australia and Arctic Russia. *Aust. J. Zool.* **62**, 26–43, doi:10.1071/ZO13081 (2014).
8. Stensiö, E. A. On the heads of certain arthrodires. 2. On the cranium and cervical joint of the Dolichothoraci. *K. Sven. Vetensk. Akad. Handl.* **22**, 1–70 (1945).
9. Hills, E. S. On certain endocranial structures in *Coccosteus*. *Geol. Mag.* **73**, 213-226, doi:https://doi.org/10.1017/S0016756800097399 (1936).
10. White, E. I. & Toombs, H. A. The buchanosteid arthrodires of Australia. *Bull. Brit. Mus. (Nat. Hist.), (Geol.)* **22**, 379–419 (1972).
11. White, E. I. Australian arthrodires. *Bull. Brit. Mus. (Nat. Hist.), (Geol.)* **1**, 249–304 (1952).
12. Mark-Kurik, E. & Young, G. C. A new buchanosteid arthrodire (placoderm fish) from the Early Devonian of the Ural Mountains. *J. Vertebr. Paleontol.* **23**, 13–27 (2003).
13. Lelièvre, H. *et al.* Nouveaux Vertébrés (Placodermes, Acanthodiens, Chondrichthyens et Sarcoptérygiens) de la formation de Jauf (Dévonien inférieur, région de Al Huj, Arabie Saoudite). *Compt. Rend. Sci., Ser. II* **319**, 1247–1254 (1994).
14. Wang, J.-Q. New material of *Buchanosteus* and its biostratigrapic significance. *Geol. Bull. Chin.* **24**, 800–806 (In Chinese) (2005).
15. Denison, R. H. in *Handbook of Paleoichthyology, Vol. 2* (ed. Schultze, H-P) 1–128 (Gustav Fischer, 1978).
16. Goujet, D. F. *Les poissons placodermes du Spitsberg. Arthrodires Dolichothoraci de la Formation de Wood Bay (Dévonien inférieur)*. Vol. 15 (CNRS, 1984).
17. Jarvik, E. *Basic Structure and Evolution of Vertebrates, Vol. 1*. (Academic Press, 1980).

18 Giles, S., Friedman, M. & Brazeau, M. D. Osteichthyan-like cranial conditions in an Early Devonian stem gnathostome. *Nature* **520**, 82–85, doi:10.1038/nature14065 (2015).

19 Stensiö, E. A. Anatomical studies on the arthrodiran head. Part 1. Preface, geological and geographical distribution, the organization of the head in the Dolichothoraci, Coccosteomorphi and Pachyosteomorphi. Taxonomic appendix. *K. Sven. Vetensk. Akad. Handl.* **9**, 1–419 (1963).

20 Goujet, D. F. in *Problèmes actuels de Paléontologie-Evolution des Vertébrés* Vol. 218 (ed. Lehman, J. P.) 81–99 (CNRS, 1975).

21 Dupret, V., Sanchez, S., Goujet, D., Tafforeau, P. & Ahlberg, P. E. A primitive placoderm sheds light on the origin of the jawed vertebrate face. *Nature* **507**, 500–503, doi:10.1038/nature12980 (2014).

22 Young, G. C. The relationships of placoderm fishes. *Zool. J. Linn. Soc.* **88**, 1–57 (1986).

23 Gardiner, B. G. & Miles, R. S. A new genus of eubrachythoracid arthrodire from Gogo, Western Australia. *Zool. J. Linn. Soc.* **99**, 159–204 (1990).

24 Dennis-Bryan, K. A new species of eastmanosteid arthrodire (Pisces: Placodermi) from Gogo, Western Australia. *Zool. J. Linn. Soc.* **90**, 1–64 (1987).

25 Ørvig, T. in *Problèmes actuels de Paléontologie-Evolution des Vertébrés* Vol. 218 (ed. Lehman, J. P.) 41–71 (Colloques Internationaux du Centre National de la Recherche Scientifique, 1975).

26 Schaeffer, B. in *Problèmes actuels de Paléontologie-Evolution des Vertébrés* Vol. 218 (ed. Lehman, J. P.) 101–109 (Colloques Internationaux du Centre National de la Recherche Scientifique, 1975).

27 Miles, R. S. The Holonematidae (placoderm fishes), a review based on new specimens of *Holonema* from the Upper Devonian of western Australia. *Philos.Trans.R.Soc.London, Ser.B* **263**, 101–234 (1971).

28 Goujet, D. F. & Young, G. C. in *Recent Advances in the Origin and Early Radiation of Vertebrates* (eds. Arratia, G. Wilson, M. V. H. & Cloutier, R.) 109–126 (Verlag Dr. Friedrich Pfeil, 2004).

29 Burrow, C. J. Placoderm fauna from the Connemarra Formation (?late Lochkovian, Early Devonian), central New South Wales, Australia. *Alcheringa* (*Special Issue 1), Suppl to vol.* **30**, 59–88 (2006).

30 Zangerl, R. in *Handbook of Paleoichthyology. Volume 3A: Chondrichthyes I. Paleozoic Elasmobranchii.* (ed. Schultze, H-P) 1–115 (Gustav Fischer, 1981)

31 Long, J. A. *et al*. First Shark from the Late Devonian (Frasnian) Gogo Formation, Western Australia Sheds New Light on the Development of Tessellated Calcified Cartilage. *PLoS One* **11**: e0126066. https://doi.org/10.1371/journal.pone.0126066 (2015).

32 Young, G. C. A new Early Devonian placoderm from New South Wales, Australia, with a discussion of placoderm phylogeny. *Palaeontogr. Abt. A* **167**, 10–76 (1980).

33 Jarvik, E. Middle and Upper Devonian Porolepiformes from East Greenland with special reference to *Glyptolepis groenlandica* n. sp., and a discussion on the structure of the head in the Porolepiformes. *Meddr. Grønland.* **187**, 1–307 (1972).

34 Allis, E. P. The cranial anatomy of *Chlamydoselachus anguineus*. *Acta Zool.* **4**, 123–221 (1923).
